# miR160 Interacts *in vivo* With *Pinus pinaster AUXIN RESPONSE FACTOR 18* Target Site and Negatively Regulates Its Expression During Conifer Somatic Embryo Development

**DOI:** 10.3389/fpls.2022.857611

**Published:** 2022-03-15

**Authors:** Ana Alves, Ana Confraria, Susana Lopes, Bruno Costa, Pedro Perdiguero, Ana Milhinhos, Elena Baena-González, Sandra Correia, Célia M. Miguel

**Affiliations:** ^1^Faculty of Sciences, BioISI—Biosystems and Integrative Sciences Institute, University of Lisbon, Lisbon, Portugal; ^2^Instituto Gulbenkian de Ciência, Oeiras, Portugal; ^3^GREEN-IT Bioresources for Sustainability, ITQB NOVA, Oeiras, Portugal; ^4^INESC-ID, Instituto Superior Técnico, Universidade de Lisboa, Lisbon, Portugal; ^5^Department of Genetics, Physiology and Microbiology, Faculty of Biological Sciences, Complutense University of Madrid (UCM), Madrid, Spain; ^6^Department of Life Sciences, Centre for Functional Ecology, University of Coimbra, Coimbra, Portugal; ^7^Instituto de Biologia Experimental e Tecnológica, Oeiras, Portugal

**Keywords:** microRNA, *ARF*, pine, *Pinus pinaster*, gymnosperm, embryogenesis

## Abstract

MicroRNAs (miRNAs) are key regulators of several plant developmental processes including embryogenesis. Most miRNA families are conserved across major groups of plant species, but their regulatory roles have been studied mainly in model species like *Arabidopsis* and other angiosperms. In gymnosperms, miRNA-dependent regulation has been less studied since functional approaches in these species are often difficult to establish. Given the fundamental roles of auxin signaling in somatic embryogenesis (SE) induction and embryo development, we investigated a previously predicted interaction between miR160 and a putative target encoding *AUXIN RESPONSE FACTOR 18* in *Pinus pinaster* (*PpARF18*) embryonic tissues. Phylogenetic analysis of *AUXIN RESPONSE FACTOR 18* (*ARF18*) from *Pinus pinaster* and *Picea abies*, used here as a model system of conifer embryogenesis, showed their close relatedness to *AUXIN RESPONSE FACTOR* (*ARF*) genes known to be targeted by miR160 in other species, including Arabidopsis *ARF10* and *ARF16*. By using a luciferase (LUC) reporter system for miRNA activity in *Arabidopsis* protoplasts, we have confirmed that *P. pinaster* miR160 (ppi-miR160) interacts *in vivo* with *PpARF18* target site. When the primary miR160 from *P. pinaster* was overexpressed in protoplasts under non-limiting levels of ARGONAUTE1, a significant increase of miR160 target cleavage activity was observed. In contrast, co-expression of the primary miRNA and the target mimic *MIM160* led to a decrease of miR160 activity. Our results further support that this interaction is functional during consecutive stages of SE in the conifer model *P. abies*. Expression analyses conducted in five stages of development, from proembryogenic masses (PEMs) to the mature embryo, show that conifer *ARF18* is negatively regulated by miR160 toward the fully developed mature embryo when miR160 reached its highest expression level. This study reports the first *in vivo* validation of a predicted target site of a conifer miRNA supporting the conservation of miR160 interaction with *ARF* targets in gymnosperms. The approach used here should be useful for future characterization of miRNA functions in conifer embryogenesis.

## Introduction

MicroRNAs (miRNAs) are small non-coding RNA molecules (20–24 nucleotides) involved in the regulation of gene expression in all domains of life, including in plants, mammals, and even in bacteria and viruses. In plants, *MIR* genes are transcribed by RNA polymerase II into primary miRNAs (pri-miRNAs). Due to the presence of complementary regions within the transcribed sequences, hairpin structures are formed and processed by the RNase III-type endonuclease DICER-LIKE 1 to form precursor miRNAs (pre-miRNAs). The pre-miRNAs undergo additional processing by DCL1 and its partners HYPONASTIC LEAVES 1 and SERRATE, producing miRNA/miRNA* duplexes, which are then loaded into ARGONAUTE1 (AGO1). AGO1 is the effector protein within the RNA-Induced Silencing Complex (RISC), directing translational inhibition or cleavage of the target mRNA transcripts by sequence complementarity of the loaded miRNA (reviewed by Achkar et al., 2016; Yu et al., 2017).

MicroRNAs regulate a broad range of biological processes, such as apoptosis, metabolism, development, and cell proliferation (Ambros, 2004; Bartel, 2004). Since plant miRNAs and their involvement in developmental processes were first reported in *Arabidopsis* (Reinhart et al., 2002), several studies over the last years have uncovered fundamental functions of miRNAs in plant embryo patterning and maturation (Schwartz et al., 1994; Nodine and Bartel, 2010; Willmann et al., 2011; Seefried et al., 2014; Plotnikova et al., 2019). In fact, miRNAs and their associated machinery are so essential that disrupting the miRNA multiprotein regulatory system or effector proteins in higher plants leads to embryo development arrestment at the early globular stage (Schwartz et al., 1994). The functions of plant miRNAs during embryogenesis; however, remain poorly characterized mainly due to the small size of early zygotic embryos embedded in maternal seed coat tissues, making their isolation and subsequent characterization of the RNA populations a difficult task (Vashisht and Nodine, 2014; Schon and Nodine, 2017). Therefore, somatic embryogenesis (SE), in which somatic cells are induced to undergo embryogenic transition, further progressing to the development of embryos that mirror their zygotic counterparts, is widely used as an experimental model to study zygotic embryogenesis (ZE).

Transcriptomic analysis in *Arabidopsis* revealed that about 98% of *MIR* genes, from 114 families, are active during SE induction (Szyrajew et al., 2017). In addition, miRNAs control of transcription factors and phytohormone metabolic pathways is key during different steps of the SE process (reviewed in Siddiqui et al., 2019). As in *Arabidopsis*, other angiosperms like *Oryza sativa*, *Zea mays*, *Gossypium hirsutum*, *Solanum lycopersicum*, and *Dimocarpus longan*, show differential expression of miRNAs at several developmental stages of SE (reviewed by Alves et al., 2021). In gymnosperms, on the other hand, not many miRNA studies are reported. A genome-wide transcriptomic study conducted in *Pinus pinaster* provided data suggesting a relevant role of miRNAs during zygotic embryo development (de Vega-Bartol et al., 2013). In *Larix laptolerix* (Zhang et al., 2012, 2013), *Picea balfouriana* (Li et al., 2017), and *P. pinaster* (Rodrigues et al., 2019), miRNA expression profiles also point to important miRNA regulatory functions during SE. Particularly in *P. pinaster*, 36 conserved miRNAs from 17 miRNA families were found differentially expressed during zygotic embryo development (Rodrigues et al., 2019). Among these, several miR160 isoforms were upregulated in late zygotic and late somatic embryos (Rodrigues et al., 2019). MiR160 is also known to be involved in *Arabidopsis* embryogenesis, being associated to the regulation of auxin signaling by targeting several *AUXIN RESPONSE FACTORS* (*ARFs*), namely *ARF10*, *ARF16*, and *ARF17* (Rhoades et al., 2002; Liu et al., 2007, 2020; Wójcik et al., 2017). Repression of *ARF17* by miR160 during *Arabidopsis* ZE was shown to be required for proper subprotodermal cell division patterns (Liu et al., 2007; Plotnikova et al., 2019). The opposite expression profiles of *ARF10/ARF16/ARF17* and miR160 in *Arabidopsis* embryogenic cultures suggested that miR160 might also contribute to the acquisition of embryogenic capacity in *Arabidopsis* somatic cells (Szyrajew et al., 2017; Wójcik et al., 2017). Direct repression of both *ARF10* and *ARF17* by miR160 regulates the development of the root cap, the activity of the root apical meristem (RAM; Wang et al., 2005), hypocotyl elongation (Dai et al., 2021), root architecture, and seed germination (Liu et al., 2007). Also in *Arabidopsis*, miR160-*ARF10* were linked to the control of cellular reprogramming and callus formation (Liu et al., 2016). In *P. pinaster*, one of the miR160 isoforms was upregulated in late zygotic and mature somatic embryos (Rodrigues et al., 2019) and it putatively targets a transcript annotated as *ARF18* (*AUXIN RESPONSE FACTOR 18* in *Pinus pinaster*, *PpARF18*; Cañas et al., 2017).

Despite the apparent conservation between most miRNA families in angiosperms and gymnosperms (Zhang et al., 2006), the regulatory interaction between miRNAs and target genes has been barely explored in gymnosperms because functional approaches are more difficult to establish in these plants. In this work, we addressed the functional conservation of miR160 in the regulation of auxin signaling during conifer embryogenesis. Firstly, we validated *PpARF18* as a target of *P. pinaster* miR160 (ppi-miR160) using a reporter system for miRNA activity in *Arabidopsis* protoplasts. Secondly, we analyzed the expression patterns of miR160 and its validated target in consecutive stages of embryo development as a first step toward their functional characterization. As a model system for conifer embryogenesis, we used Norway spruce (*Picea abies*; Filonova et al., 2000; von Arnold and Clapham, 2008) due to its well-established and highly synchronized system of embryo development.

## Materials and Methods

### Plant Material and Growth Conditions

*Arabidopsis thaliana* (L.) Heynh. plants in Columbia (Col-*0*) background were used to validate the miRNA-target interaction. Sterilized and stratified seeds of wild-type (WT) and the transgenic lines *mir160b* and *mir160c* (Wójcik et al., 2017) were sowed in pots with a 1:3 vermiculite/soil mixture. Plants were grown under a photoperiod of 12 h light (100 μE; 22°C)/12 h dark (18°C). Leaves of 5-week-old plants were harvested 2 h after the onset of the light period, both for protoplasts isolation and for RNA extraction.

The embryogenic cell line 61:21 of Norway spruce (*P. abies* L. Karst) was used as a model system for conifer somatic embryogenesis. The terminology used to describe somatic embryogenesis at different SE developmenral stages in this report was based on the referenced articles (Filonova et al., 2000; von Arnold and Clapham, 2008). The cultures were treated as described previously (von Arnold and Clapham, 2008). Briefly, proembryogenic masses (PEMs) were maintained every 2–3 weeks under proliferation on half-strength LP medium (von Arnold and Eriksson, 1981) supplemented with 9 μM 2,4-D, 4.4 μM BA, and 1% sucrose. To stimulate differentiation of early somatic embryos (EEs), cultures were washed by transferring 4–5 embryogenic clusters to tubes containing 50 ml half-strength liquid LP medium (prematuration medium). After washing by slowly inverting the tube for 1 min, settled cell aggregates (approx. 5 ml) were transferred to 250 ml Erlenmeyer flasks with 100 ml of prematuration medium. The cultures were grown on a gyratory shaker at 100 rpm, in darkness at 22°C. After 1 week, 2 ml of suspension cells were plated on top of two filter papers (Whatman no. 2) placed on maturation medium BMI-SI (Krogstrup, 1986) supplemented with 30 μM abscisic acid (ABA) and 3% sucrose, for the development of late embryos (LEs) and mature embryos (MEs). The filter papers were transferred to fresh maturation medium every 2 weeks and kept in the dark at 22°C. The media were solidified with 0.35% (w/v) Gelrite and the pH was adjusted to 5.8 before autoclaving. For the maturation medium, L-Glutamine (3 mM) and ABA were filter-sterilized and added to the autoclaved and cooled medium prior to pouring into sterile Petri dishes. To study the expression profiles of miR160 and *ARF18* during *P. abies* embryo development, 70–100 mg of PEMs, EEs, LEs, and MEs samples were collected for RNA extraction. PEMI and PEMIII were collected as cell aggregates from the proliferation medium. EEs and LEs were collected as cell aggregates after 1 week on prematuration medium and after 2–3 weeks on maturation medium, respectively. ME developed after 5–8 weeks on maturation medium. After 5 weeks ME1 were collected as maturing embryos characterized by the initiation of cotyledons. After 6–7 and 8 weeks, incompletely mature (ME2) and fully mature (ME3) embryos with at least four cotyledons were collected, respectively. The samples were frozen in liquid nitrogen and stored at −80°C.

### *In silico* Analysis of miR160: *ARF* Interaction

The interaction between ppi-miR160 and an *ARF* encoding gene (sp_v3.0_unigene806) had been previously predicted in *Pinus pinaster* (Rodrigues et al., 2019; Perdiguero et al., 2021). To identify the homologous sequences in *P. abies* different searches were performed using nucleotide Basic Local Alignment Search Tool (BLASTN). Firstly, the precursor sequence of ppi-miR160 was used as query in miRBase database (Kozomara et al., 2019)^1^ against precursor sequences from *P. abies*. The pre-miRNA sequence in *P. abies* containing exactly the same mature miRNA sequence than *P. pinaster* was selected. Secondly, the *P. pinaster* transcript sequence encoding the *ARF* identified as target was used as query in ConGenie database against high confidence gene models from *P. abies*. Homologous sequences to the conifer mature miR160 were also searched in well-documented model species, including *Arabidopsis thaliana*, *Oryza sativa*, and *Solanum lycopersicum*. For this, data from miRBase was retrieved and sequence alignment was performed using Clustal software implemented in Jalview. To analyze the homology of conifer *ARF*s and those previously described in model species, all proteins annotated as ARFs identified in *Arabidopsis*, *O. sativa*, and *S. lycopersicum* genomes were retrieved from RefSeq database (O’Leary et al., 2016)^2^ and aligned with deduced amino acid sequences from conifer *ARF*s using ClustalW implemented in MEGA software. The phylogenetic tree was generated using the maximum-likelihood ratio method and tested by using bootstrap with 1,000 replications.

### Cloning and Preparation of Constructs for *Arabidopsis* Protoplasts Transfection

The pUC18-based pHBT95 (accession no. EF090408; Yoo et al., 2007) was used as backbone for the constructs used in protoplast transfection. This included reporter constructs (Supplementary Figure S1A) expressing the firefly luciferase (*fLUC*) gene and constructs (Supplementary Figure S1B), where effectors were expressed under the *35S* promoter and *NOS* terminator (Luehrsen et al., 1992).

#### Reporter Constructs

Reporters for miRNA activity were built according to the system described by Martinho et al. (2015). Specifically, the selected cleavable and noncleavable *P. pinaster* ppi-miR160 target sites were inserted in the 3′UTR of *fLUC* by site-directed mutagenesis.

*Unigene806* was identified as the putative target of *P. pinaster* ppi-miR160 (mature sequence TGCCTGGCTCCCTGTATGCCA) by Rodrigues et al. (2019) when using psRNAtarget (Dai and Zhao, 2011; Dai et al., 2018) against the reference *P. pinaster* transcriptome (Canales et al., 2014). In the latest version of the *P. pinaster* transcriptome, *Unigene806* is annotated as *ARF18* (*PpARF18*; Cañas et al., 2017).

To build a specific luciferase (LUC)-based reporter for ppi-miR160 activity, we introduced the putative target site from *Unigene806* in the 3′UTR of luciferase by site-directed mutagenesis, similarly to what was described by Martinho et al. (2015)—primer pair LUC_C_ARF18 (Supplementary Table S1). In parallel to this cleavable version (C), we generated a non-cleavable construct (NC) as control—primer pair LUC_NC_ARF18 (Supplementary Table S1). Primers for NC reporter carried two mutations in the complementary position 10 and 11 of the ppi-miR160. The PCR reaction for mutagenesis consisted of 2.5 μl *Pfu* buffer, 2.5 μl 2.5 mM dNTPs, 1.5 μl of 1 μM of each primer (Supplementary Table S1), 50 ng of plasmid DNA pHBT95, 0.5 μl *Pfu* DNA Polymerase (Promega), and sterile water up to 25 μl. The reaction mix was split into two 12.5 μl aliquots: one used for PCR amplification and a second one kept as a negative control. The mutagenesis PCR was carried under the following conditions: 1 cycle to 95°C for 3 min followed by 18 cycles of 30 s at 95°C, 60 s at 55 and 68°C for 10 min. Around 0.5 μl of *DpnI* was added to both PCR reaction and the negative control and then incubated at 37°C overnight. About 4 μl of each reaction was used to transform 50 μl of MC1061 competent cells. The constructs were verified by sequencing.

For growing cultures for plasmid DNA maxipreps, *Escherichia coli* MC1061 were used to achieve a consistently high DNA yield and quality. Bacteria transformation, growth and plasmid isolation, and purification were performed as described (Confraria and Baena-González, 2016).

#### Effector Constructs

Primers were designed containing the appropriate restriction sites (BamHI and PstI) to amplify and clone the miR160 precursor downstream of the constitutive *35S* promoter. For amplification of pri-miR160 (pri160) primers were designed based on *Pinus taeda* genomic sequences, scaffold C32559718, position 66,547–66,567 (Rodrigues et al., 2019) encompassing 200 bp upstream and downstream of the 5′ and 3′ of the mature miRNA (Cuperus et al., 2010; Supplementary Table S1, primiR160_BamHI_F and primiR160_PstI_R) and amplification was performed from *P. pinaster* genomic DNA isolated from root tissues with the CTAB method (Doyle and Doyle, 1990). The pri160 sequence was PCR amplified with 0.2 mM of dNTPs, 0.5 μM of each primer, 0.02 U/μl of Phusion™ HF DNA Polymerase (Thermo Scientic™), 1x Phusion HF Buffer, and 50–250 ng of gDNA. The cycling conditions consisted of 30 s at 98°C, followed by 35 cycles of 10 s at 98°C, 20 s at 55°C as T annealing and 2 min at 72°C, and the last cycle of 5 min at 72°C. The PCR products were separated by electrophoresis in 0.8% agarose gel stained with RedSafe™ Nucleic Acid Staining Solution (iNtRON Biotechnology) and visualized under UV light. PCR products were purified using High Pure PCR Product Purification Kit (Roche), cloned into pGEM®-T Easy (Promega) which was then transformed into JM109 High-Efficiency Competent Cells, following manufacturer’s instructions. The transformants with confirmed insert by colony PCR were grown overnight at 37°C in liquid LB supplemented with 100 μg/ml ampicillin and then the plasmid DNA was extracted using the QIAprep Spin Miniprep Kit (QIAGEN), according to the manufacturer’s instructions. After sequence confirmation by Sanger sequencing, double digestion with BamHI (MB09201, NZYTECH) and PstI (MB10301, NZYTECH) of the plasmid with the insert (pri160) and the vector pHBT95 was performed. Both insert and vector digestion products were separated by electrophoresis in agarose gel and purified using the High Pure PCR Product Purification Kit (Roche). The purified insert was ligated to pHBT95 using T4 Ligase (MB000703, NZYTECH), and then transformed into MC1061 competent cells.

To generate *MIM160*, site-specific mutagenesis was performed using a *MIM319* construct (Martinho et al., 2015) as template. The PCR reaction conditions for site-directed mutagenesis were the same as described above, using the primers IPS1_MIM160 (Supplementary Table S1).

### *Arabidopsis* Protoplast Isolation and Transfection

Fully expanded leaves of 5-week-old non-flowering plants were used for protoplast isolation as already described (Confraria and Baena-González, 2016).

For miRNA activity assays, 8–10 μg of luciferase-based reporters were used in combination with 12–10 μg of effector/control constructs and 1 μg of *35S::GUS* (Martinho et al., 2015) as transfection control. All the constructs used, including the controls, are listed in Table 1. About 2 × 10^4^ protoplasts were transfected using a ratio of 1 μg CsCl-purified maxiprep plasmid DNA per 1 × 10^3^ transfected protoplasts. The mER7 plasmid was used as control DNA (Kovtun et al., 1998). After PEG-Ca^2+^ transfection (Confraria and Baena-González, 2016) protoplasts were incubated overnight under light (15 μE, 25°C). On the following day, protoplasts were harvested by centrifugation at 100 *g* for 3 min, flash-frozen on dry ice, and used for luciferase and β-glucuronidase analysis (Confraria and Baena-González, 2016). To calculate normalized relative light units (nRLU), LUC activity values were divided by GUS activity values. Four biological replicates, corresponding to four independent protoplast batches, and each replicate consisting of two independent transfections were used to calculate the mean and SE for each sample.

**Table 1:** List of the plasmids used in protoplast transfection.

Vector	Insert	Resistance	Description
pHBT95	mER7	Ampicillin	Control DNA
pHBT95	GUS	Ampicillin	Transfection control
p35S-HA-GW	AGO1	Ampicillin	WT AGO1, HA-tagged
pHBT95	pri160	Ampicillin	Genomic sequence for primary ppi-miR160
pHBT95	*MIM160*	Ampicillin	*IPS1* containing target mimic for ppi-miR160
pHBT95	C-*fLUC_ARF18_*	Ampicillin	Target site for ppi-miR160 (from *PpARF18*) introduced into 3′UTR of firefly luciferase
pHBT95	NC-*fLUC_ARF18_*	Ampicillin	Non-cleavable target site for ppi-miR160 (mutated from *PpARF18*) introduced in 3′UTR of firefly luciferase

### RNA Extraction

Total RNA was extracted from Norway spruce embryonic tissues using the Plant/Fungi Total RNA Purification Kit (NORGEN BIOTEK CORP.), according to kit instructions. To eliminate DNA contamination, the rigorous protocol of DNase TURBO*-free*™ Kit (Invitrogen) was used. RNA samples were quantified using the fluorometer Qubit 3.0 using RNA BR Assay Kit (Thermo Fisher Scientific).

### Quantitative RT-qPCR

For quantifying miR160 expression an absolute quantification was performed using ready to order TaqMan® MicroRNA Assay ID 000341 for ath-miR160a (Applied Biosystems® by Thermo Fisher Scientific) using total RNA from seven different stages of Norway spruce embryo development (PEMI, PEMIII, EE, LE, ME1, ME2, and ME3). Oligonucleotides identical to the conserved mature miR160 were ordered^3^ and used to prepare a standard curve. The complementary DNA (cDNA) synthesis was performed from 10 ng of DNase treated total RNA using TaqMan® MicroRNA Reverse Transcription Kit (Applied Biosystems® by Thermo Fisher Scientific) and the miRNA-specific 5x RT primer provided. Each 20 μl qPCR reaction mixture included 1x TaqMan® Universal PCR Master Mix II, No UNG (Applied Biosystems®), 1x TaqMan® MicroRNA Assay primer 20x and the cDNA, prepared according to manufacturer’s recommendations.

Relative quantification of *ARF18* in *P. abies* (MA_98506g0010)^4^ was performed in the same stages of Norway spruce embryo development mentioned above. Primers (RT_pabARF18) were designed using Primer3web (Supplementary Table S1).^5^ cDNA synthesis was performed using SuperScript® IV First-Strand Synthesis System (Invitrogen™) and oligo(dT)_20_ primer using 1 μg of total RNA per 13 μl reaction, according to manufacturer’s instructions. Each 20 μl qPCR reaction mix included 1x SYBR Green I Master (Roche Diagnostics), 500 nM of each primer and 1,5 μl of 1:5 diluted cDNA. The amplification program was the same for all genes: 95°C for 5 min, 40 cycles of 10 s at 95°C, 15 s at 62°C, and 12 s at 72°C. Primer specificity was monitored by analyzing the melting curves. Negative controls were prepared using total RNA as template in qPCR amplification. As additional controls, positive and non-template controls (NTC) were included in all plates. *Picea abies ARF18* transcript profiles were normalized using three reference genes *CDC2* (Wadenbäck et al., 2008), *EF-α*, and *PHOS* (Vestman et al., 2011). Relative expression levels were calculated using the Pfaffl (2001) method.

All qPCR experiments were performed in a LightCycler 480 (Roche Diagnostics) with white 96-well plates (Roche Diagnostics). The experiments included three biological replicates with at least two technical replicates, except for EEs for which two biological replicates were used.

### Statistical Analysis

The statistical analyses were performed with Prism 9 program using either the unpaired Student *t*-test or a two-way ANOVA (*p* < 0.05), followed by Tukey’s honestly significant difference test (Tukey HSD-test; *p* < 0.05) or Sidak’s multiple comparison test. The figures show the average from biological replicates with the SE or SD.

## Results

### Conifer *ARF18* Is Closely Related to *Arabidopsis ARF10/ARF16*

**Figure 1:**
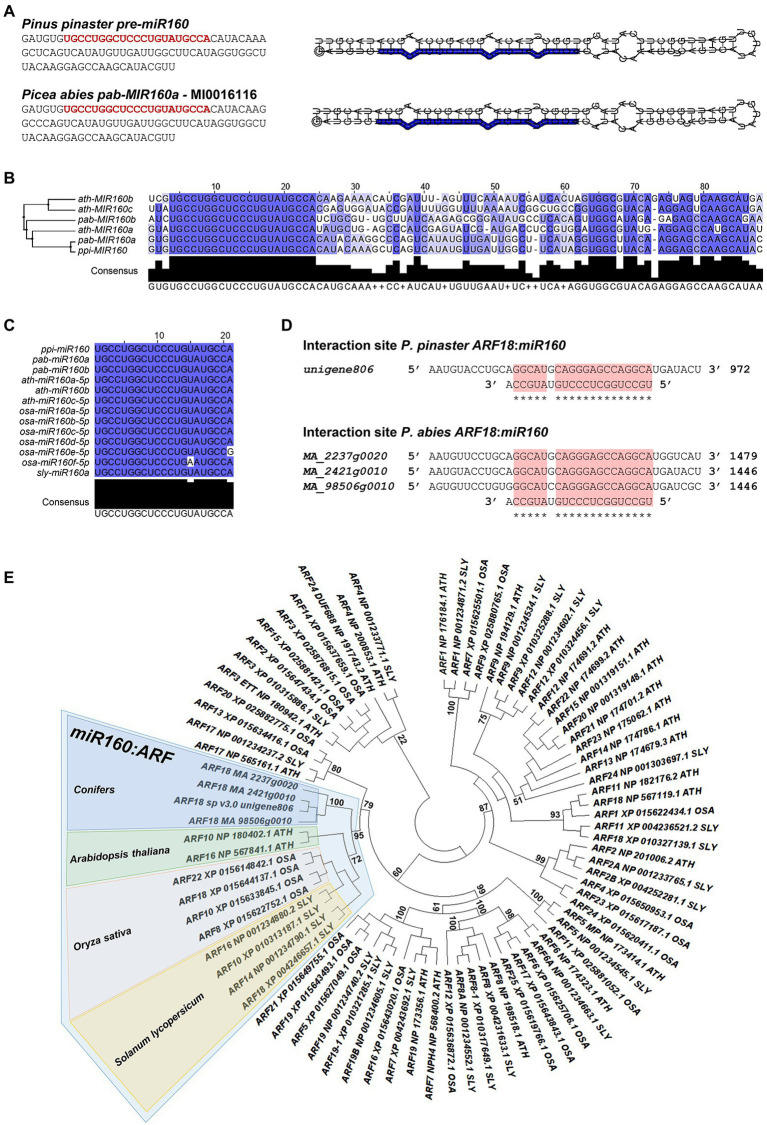
*In silico* analysis of miR160: *AUXIN RESPONSE FACTOR* (*ARF*) interaction. **(A)** *Pinus pinaster* and *Picea abies* precursor-miR160 (pre-miR160) sequences. The sequence from *P. pinaster* was obtained in this work, whereas the *P. abies* sequence corresponds to MI0016116, annotated as pab-MIR160a in miRBase database. The miR160 mature sequence is highlighted in red. The shown secondary structures for both precursors were predicted and visualized using RNA Folding annotation tool implemented in the UEA sRNA workbench (Stocks et al., 2012). **(B)** Sequence alignment and phylogenetic tree constructed by the Maximum-Likelihood method of miR160 precursor sequences from *Arabidopsis thaliana* (*ath*), *P. abies* (*pab*) and *P. pinaster* (*ppi*). **(C)** Alignment of mature miR160 sequences from *A. thaliana* (*ath*), *Oryza sativa* (*osa*), *Solanum lycopersicum* (*sly*), *P. abies* (*pab*), and *P. pinaster* (*ppi*). High sequence conservation is represented in dark blue; white positions indicate no sequence conservation. **(D)** Target sites for miR160 in *P. pinaster* and *P. abies* transcripts annotated as *AUXIN RESPONSE FACTOR 18* (*ARF18*). The target site is indicated with a red box. **(E)** Phylogenetic tree of ARF protein sequences from *A. thaliana*, *O. sativa*, and *S. lycopersicum* and deduced amino acid sequences of *ARF18* identified in conifers as potential target for miR160. The highlighted branch shows the more correlated proteins between the different species. The maximum-likelihood method was used with 1,000 bootstrap replicates.

To investigate the interaction of ppi-miR160 with its predicted target, we started by confirming the genomic sequence of the precursor (pre-miR160) through PCR amplification and sequencing (Figure 1A). Since *P. abies* SE was used in this work as a model for analysis of miR160-*ARF18* expression during embryo development, a BLASTN search was performed against *P. abies* precursor sequences available in miRBase resulting in the identification of pab-*MIR160*a (accession MI0016116). The mature sequence within pab-*MIR160*a is identical to ppi-*MIR*160 and only two mismatches were found between the precursor sequences (Figure 1B), which have no influence in the secondary structure (Figure 1A). The alignment performed with *Arabidopsis* miR160 precursor sequences revealed that ath-*MIR160*a is the closest to ppi-*MIR160* and pab-*MIR160*a (Figure 1B). As to the mature miR160, alignment of *P. pinaster* and *P. abies* sequences with those from *Arabidopsis*, *O. sativa*, and *S. lycopersicum* available from miRbase (Kozomara et al., 2019) showed that they are identical, with the exception of *Osa-miR160e* and *Osa-miR160f* (Figure 1C).

In previous reports of *P. pinaster* miRNA analyses, *Unigene806* was identified as a potential target of ppi-miR160 in developing embryos (Rodrigues et al., 2019) and in roots from adult plants by degradome analysis (Perdiguero et al., 2021). A BLASTN search using *P. pinaster Unigene806* as query against Congenie database (v1.0; Nystedt, et al., 2013; Sundell et al., 2015) resulted in the identification of three high confidence gene models in *P. abies* (*MA_2237g0020*, *MA_2421g0010* and *MA_98506g0010*) showing high coverage of the *P. pinaster* sequence. All these transcripts are annotated as *AUXIN RESPONSE FACTOR* 18 (*ARF18*) both in the last version of *P. pinaster* transcriptome (Cañas et al., 2017; de Maria et al., 2020; Modesto et al., 2021) and in Congenie database (accessed data December 2021), and all the sequences show a potential target site for miR160 (Figure 1D). The phylogenetic analysis performed with *P. pinaster* and *P. abies ARF18* sequences to determine their relationship with the *ARF*s from other selected species highlighted a global branch that grouped them with *Arabidopsis* (*Ath*) *ARF10* and *ARF16*, *O. sativa* (*Osa*) *ARF8*, *ARF10*, *ARF18*, and *ARF22*, and also *ARF10*, *ARF14*, *ARF16*, and *ARF18* from *S. lycopersicum* (*Sly*; Figure 1E).

### *PpARF18* Cleavable Reporter Is Recognized by ath-miR160a

**Figure 2:**
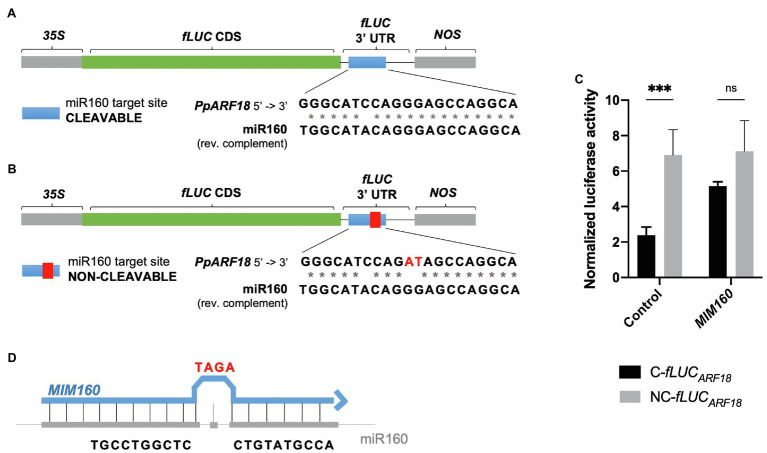
Firefly Luciferase (*fLUC*) microRNA (miRNA) reporters used to monitor miR160 activity in *Arabidopsis* protoplasts. **(A)** The *P. pinaster* miR160 (ppi-miR160) *AUXIN RESPONSE FACTOR 18* in *Pinus pinaster* (Pp*ARF18*) target site was introduced in the 3′UTR region of *fLUC*, generating the cleavable reporter. **(B)** The non-cleavable reporter was produced harboring mutations in positions corresponding to the 10th and 11th nucleotides of the mature miR160 sequence (showed in red). **(C)** Normalized luciferase activity of cleavable (C*-fLUC*) and noncleavable (NC*-fLUC*) reporters as a measure of miR160a activity. Asterisks (*) represent significant differences (*p* < 0.001) obtained by two-way ANOVA and Sidak’s multiple comparison test. **(D)** Target mimic with a modification of the central sequence by addition of three extra nucleotides (C to TAGA), resulting in a bulge formation.

By combining miRNA gain- and loss-of-function through miRNA and target mimics overexpression, we probed the *in vivo* interaction of *P. pinaster* miR160 with its predicted *PpARF18* target site (Rodrigues et al., 2019). To this aim, *fLUC*-based miRNA activity sensors were generated for transient expression assays using *Arabidopsis* mesophyll protoplasts. We started by constructing a reporter with the putative target cleavage site sequence in the 3′UTR of *fLUC* (“cleavable” C-*fLUC_ARF18_*—Figure 2A). In parallel, we used site-directed mutagenesis to engineer a noncleavable version carrying a target site with mutations in positions complementary to positions 10 and 11 of ppi-miR160 to prevent slicing (“noncleavable” NC-*fLUC_ARF18_*; Figure 2B; Li et al., 2013; Martinho et al., 2015). As a first test, the *fLUC*_ARF18_ reporters were transiently expressed in *Arabidopsis* protoplasts. Given that mature miR160 sequences in both *Arabidopsis* and *P. pinaster* were found identical (Figure 1C), we expected the miRNA target site in the C-*fLUC_ARF18_* would be recognized by the endogenous miRNA (ath-miR160), thus modulating target expression through mRNA cleavage. After determining the normalized luciferase activity, considered as an inverse quantitative readout of miRNA activity, a significantly lower activity was obtained for the C-*fLUC_ARF18_* when compared to its noncleavable variant (Control, Figure 2C). These results are consistent with a high ath-miR160 activity, confirming the susceptibility of the cleavable *fLUC_ARF18_* reporter to endogenous ath-miR160 post-transcriptional regulation.

To further test the specificity of the miRNA reporters, a target mimic was overexpressed (*MIM160*) to downregulate miR160. When using target mimicry, the plant miRNA is sequestered by an RNA molecule that is only partially complementary to the miRNA, producing a bulge in the region where cleavage of the true miRNA target occurs (Figure 2D; Franco-Zorrilla et al., 2007; Todesco et al., 2010), and thus preventing miRNA function (Todesco et al., 2010). When *MIM160* was overexpressed, no significant differences were observed between the luciferase activities of the C-*fLUC_ARF18_* and NC-*fLUC_ARF18_* reporters (Figure 2C). Based on these results, we can conclude that ath-miR160 was sequestered by *MIM160*, resulting in reduced miRNA levels.

**Figure 3:**
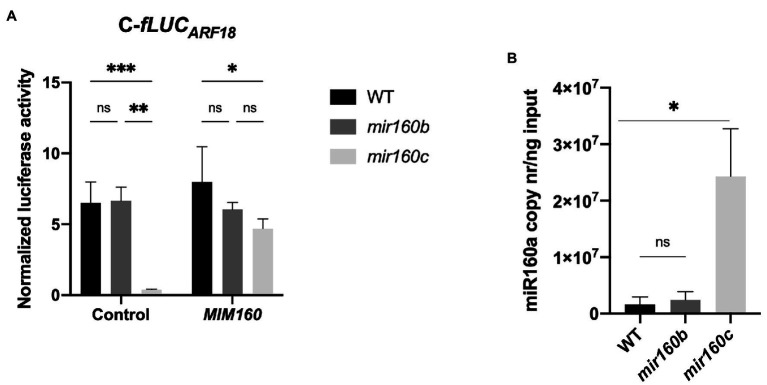
Different levels of ath-miR160a activity in *mir160b* and *mir160c Arabidopsis* protoplasts. **(A)** Normalized luciferase activity of cleavable (C-*fLUC_ARF18_*) reporter for ath-miR160a activity in wild-type (WT), *mir160b* and *mir160c Arabidopsis* protoplasts in the presence of the indicated elements. Bars represent mean ± SE of at least two independent experiments with two technical replicates each. Asterisks (*) represent significant differences according to *p* value classification (*p* < 0.05) obtained by two-way ANOVA and Tukey’s multiple comparison test. **(B)** Absolute quantification of miR160a copy number in WT vs. *mir160b* and *mir160c Arabidopsis*. Bars represent mean ± SD of three biological replicates. Asterisks (*) represent significant differences (*p* < 0.05) obtained by an unpaired *t*-test comparing each mutant to WT. ns, non-significant.

To investigate if *PpARF18* was preferentially targeted by the ath-miR160a isoform, having the most similar precursor to ppi-*MIR160* (Figure 1C), C-*fLUC_ARF18_* was expressed in *miR160b* and *miR160c Arabidopsis* loss-of-function mutants, which accumulate reduced levels of the respective isoforms (Wójcik et al., 2017). In this way, we expected any decrease in target cleavage to be the result of a lower abundance of ath-miR160, including all the isoforms. In *miR160b Arabidopsis* mutants no significant differences could be detected in the measured luciferase activity when compared to the wild-type (WT) plants (Figure 3A). Surprisingly, in *miR160c Arabidopsis* mutants a significant increase in miRNA activity was observed, as shown by the decrease in the luciferase activity between WT and the mutant (Figure 3A). Quantification of ath-miR160 in the *mir160b* and *mir160c* mutants confirmed its increased expression in *mir160c* plants (Figure 3B), possibly compensating for the *miR160c* mutation. These results further suggest that the C-*fLUC_ARF18_* reporter is preferentially targeted by the miR160a isoform.

### *Pinus pinaster* miR160 Interacts *in vivo* With Its Predicted *PpARF18* Target Site

**Figure 4:**
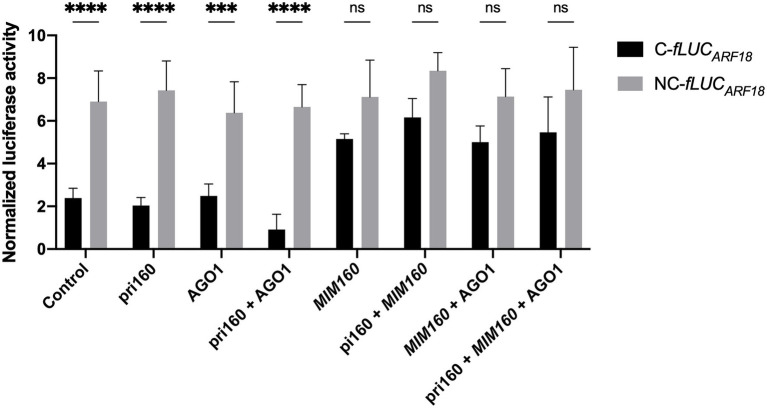
Normalized luciferase activity from reporter constructs as a measure of miR160 activity in *Arabidopsis* protoplasts transfected for overexpression of the indicated elements. Asterisks (*) represent significant differences according to *p* value classification (*p* < 0.001) obtained by two-way ANOVA and Sidak’s multiple comparison test. Bars of all graphics represent mean ± SE of four independent experiments with two technical replicates each.

Overexpressing primary miRNA sequences was shown to be sufficient for the correct processing and accumulation of the respective mature miRNAs (Martinho et al., 2015). To test the interaction of ppi-miR160 with its predicted target *PpARF18* (Rodrigues et al., 2019), the primary miR160 from *P. pinaster* (pri-miR160, or in short “pri160”) and the C-*fLUC_ARF18_* and NC-*fLUC_ARF18_* reporters were co-expressed in the presence or absence of AGO1, the effector protein in miRNA target cleavage (Achkar et al., 2016; Yu et al., 2017; Figure 4). Luciferase activity of the reporter C-*fLUC_ARF18_* was affected by endogenous ath-miR160 (Control) and showed no significant variation when pri160 and AGO1 were overexpressed alone. However, the co-expression of pri160 and AGO1 led to a decrease of 62% in luciferase activity when compared to pri160 expression, supporting the occurrence of high ppi-miR160 activity in *Arabidopsis* protoplasts when AGO1 levels are not limiting (Figure 4). Furthermore, when *MIM160* was expressed alone or in combination with other elements, the luciferase activity of the C-*fLUC_ARF18_* significantly increased, indicating a lower miR160 activity when compared to the endogenous ath-miR160 or the ppi-miR160 generated by pri160 overexpression (Figure 4). The NC-*fLUC_ARF18_* reporter was not affected by the overexpression of any other factor, remaining higher than C-*fLUC_ARF18_* in all situations. Luciferase levels of NC-*fLUC_ARF18_* reporter were identical to those obtained when *MIM160* was co-expressed with C-*fLUC_ARF18_* reporter, both indicating miR160 loss-of-function (Figure 4). These results experimentally validate the interaction between ppi-miR160 and *P. pinaster ARF18* target site, supporting *PpARF18* as a true ppi-miR160 target as predicted earlier by Rodrigues et al. (2019).

### Expression of *Picea abies ARF18* Decreases Toward More Advanced Stages of SE and Negatively Correlates With miR160 Expression

**Figure 5:**
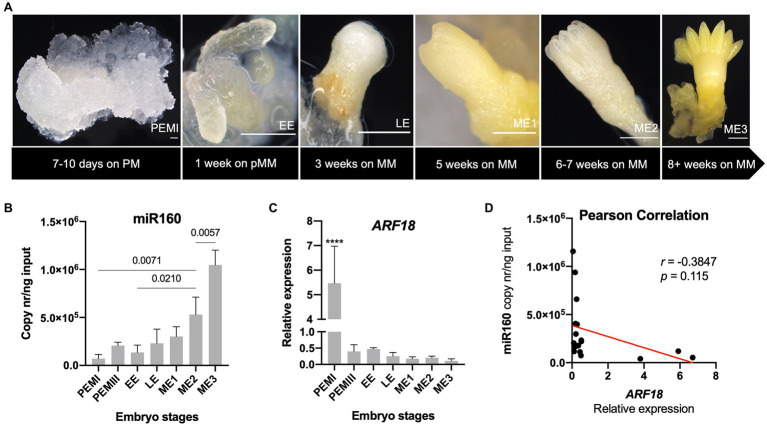
Expression profiles of *Picea abies* miR160 and *ARF18* during somatic embryogenesis. **(A)** Different developmental stages of somatic embryogenesis, from proembryogenic masses (PEMI) on proliferation medium (PM) to fully mature embryo (ME3) on maturation medium (MM). Bars correspond to 500 μm. **(B)** Absolute quantification of miR160 (copy nr/ng input) across the selected embryo developmental stages. Significant differences of pairs of bars are marked with the *p* value, obtained by one-way ANOVA and Tukey’s multiple comparison test. **(C)** Relative expression levels of *ARF18* during the selected stages of *P. abies* somatic embryo development. *CDC2*, *EF1-α*, and *PHOS* were used as reference genes. **(D)** Pearson correlation of expression levels between miR160 and *ARF18*. Error bars correspond to the SD for the RT-qPCR values of three biological replicates, except for early embryos (EEs) for which two biological replicates were used.

After validating the interaction of *PpARF18* with ppi-miR160 *in vivo*, this interaction was explored during the different stages of conifer somatic embryo development. For this, we favored using *P. abies* over *P. pinaster* due to its well-established and highly synchronized system of embryo development, which makes it an excellent model of SE in conifers (Filonova et al., 2000; von Arnold and Clapham, 2008). Also, both the ppi-miR160 isoform and the *ARF18* target sequences are conserved (Figures 1C,D) between *P. pinaster* (*Unigene806*) and *P. abies* (*MA_98506g0010*). Expression of *P. abies* miR160 and *ARF18* were evaluated in five stages of development including proembryogenic masses in proliferation (PEMs), EE, LE, and mature embryos (ME1, ME2, and ME3; Figure 5A).

The results showed that *P. abies* miR160 was strongly downregulated in the PEMI stage (Figure 5B), while the *ARF18* steady-state mRNA levels were strongly upregulated (Figure 5C). In addition, there was a progressive accumulation of miR160 in consecutive developmental stages toward *P. abies* embryo maturation, reaching its highest level in the fully developed mature embryo (ME3; Figure 5B). On the other hand, *P. abies ARF18* steady-state mRNA levels showed a clear inverse tendency, decreasing towards embryo maturation with the mature embryos presenting the lowest expression levels (Figure 5C). A negative correlation (*r* = −0.3847; *p* = 0.115) between the expression of *ARF18* and miR160 in *P. abies* SE (Figure 5D) was confirmed.

## Discussion

In this work, we showed that the miR160 of *Pinus pinaster* interacts *in vivo* with the *PpARF18* target site (Figure 4) and that this interaction is functional during somatic embryogenesis in a conifer model (Figure 5). Previous work investigating miRNAs involved in pine embryogenesis had predicted a highly probable functional miR160 binding site within *P. pinaster ARF18* target mRNA (Rodrigues et al., 2019). Computational algorithms, such as the one used, may predict numerous possible mRNA targets for a specific miRNA (Kuhn et al., 2008) but only a few will be true functional targets. In fact, *PpARF18* was among the 82 putative targets of ppi-miR160 predicted by Rodrigues et al. (2019). More recent work provided further support to the functionality of this interaction by degradome analysis in roots of *P. pinaster* (Perdiguero et al., 2021). In *Arabidopsis*, miR160 is known to target *ARF10/16/17* (Wójcik et al., 2017; Das et al., 2018; Lin et al., 2018; Dai et al., 2021) and in *P. abies*, used here as model for SE, no information is available regarding miR160 target interaction. Given that methods for *in vivo* validation of miRNA/mRNA pairs and their expression analyses during embryo development have been established in *Arabidopsis* (reporter assays in protoplasts) and *P. abies* (SE), respectively, the similarity between miR160 sequences and their respective targets in these species were analyzed and compared to the equivalent sequences in selected conifer models. Being a conserved miRNA, it was not surprising to verify that miR160 mature sequences were identical in all the analyzed species, with the exception of *Osa-miR160e* and *Osa-miR160f*. However, the precursor sequences were less similar in most cases except in *P. abies* and *P. pinaster*, where only two mismatches were detected; such mismatches did not affect the precursor secondary structure. In *Arabidopsis*, the most similar precursor corresponded to isoform ath-mirR160a, which is consistent with our transient expression results in *miR160b* and *miR160c* protoplasts (Figure 3).

As to the target sequences, our phylogenetic analysis showed that *P. pinaster* and *P. abies ARF18* grouped with *Arabidopsis AthARF10/16*, *O. sativa OsaARF8/10/18/22*, and *S. lycopersicum SlyARF10/14/16/18* (Figure 1D). From these, it is known that *AthARF10/16* (Wójcik et al., 2017; Das et al., 2018; Lin et al., 2018; Dai et al., 2021), *OsaARF8/10/18/22* (Huang et al., 2016), and *SlyARF10* (Hendelman et al., 2012) are targeted by miR160. A recent phylogenomic synteny network analysis with more than 3,500 ARFs from major streptophyte lineages proposed a classification of angiosperm *ARF* genes in six groups (Gao et al., 2020) and revealed which gymnosperms *ARF* genes were the closest sister lineage to each one of these six groups. Based on the length of the phylogeny tree branches, the authors further suggested lower amino acid substitution rates and higher levels of sequence conservation in the gymnosperm *ARFs*, possibly due to the usually longer generation times in the gymnosperms. From the three major subfamilies or clades of *ARFs* (Finet et al., 2013) encompassing the six groups, the clade C subfamily comprises genes from every major plant lineage, including the *Arabidopsis ARF10/16/17* within the group IV of angiosperms *ARF* genes (Gao et al., 2020). The split that generated angiosperms clade C *ARF10/16/17* is suggested to have occurred early in angiosperm evolution, and no duplications have been found in the ancestors of non-angiosperm species (Mutte et al., 2018; Gao et al., 2020). Our phylogenetic analysis suggests that *P. pinaster* and *P. abies ARF18*, targeted by miR160, are functionally related to the sequence that originated *ARF10* and *ARF16* in angiosperms. Thus, conifer *ARF18* might act as a putative orthologue of *AthARF10* and/or *AthARF16*.

As a first step toward the functional characterization of the miRNA-mRNA interaction during embryo development in conifers, we validated this interaction *in vivo* using a reporter system in *Arabidopsis* mesophyll protoplasts. By repressing the production of the firefly luciferase reporter protein (fLUC), we show that ppi-miR160 specifically binds to and drives the cleavage of the C-*fLUC_ARF18_* reporter, harboring the *PpARF18* target site (Figures 2, 4). While testing the sensitivity of our system to varying ath-miR160 endogenous levels using protoplasts from *miR160b* and *miR160c* mutants, we found that the ath-miR160a isoform is the most active one against the C-*fLUC_ARF18_* reporter (Figure 3). We also detected a strong increase in ath-miR160 activity in *miR160c* (Figure 3), that we attribute to the overaccumulation of ath-miR160a, as a compensatory mechanism for the *miR160c* mutation. This system for *in vivo* quantification of miRNA activity in *Arabidopsis* protoplasts (Martinho et al., 2015) revealed extremely useful to study miR160 in *P. pinaster*, which like many other gymnosperm species, are not amenable to stable transformation with miRNA reporter systems or these procedures are extremely difficult and time-consuming (Schwab et al., 2009; Nodine and Bartel, 2010; Wójcik et al., 2017). Assuming the miRNA biogenesis machinery processing the miR160 precursor sequence is conserved across both plant groups (Figure 1), and the confirmed identity of the mature miRNA160 sequences of *Arabidopsis* and *P. pinaster*/*P. abies*, it was expected that the observations in *Arabidopsis* protoplasts accurately reflect the post-transcriptional interaction occurring in the conifer’s cells.

Our results in the somatic embryogenesis of *P. abies*, where the expression of miR160 gradually increased showing the highest expression in the mature embryo stages, are in agreement with previous work in conifers (Rodrigues et al., 2019), in which the same miR160 isoform showed a higher expression in late stages of *P. pinaster* zygotic and somatic embryo development. Furthermore, and as expected by the miR160-*PpARF18* target site validated interaction, an opposite expression pattern was observed between miR160 and its target during the same developmental period (Figure 5). However, miRNA160-mediated regulation of conifer *ARF18* does not exclude its regulation by additional mechanisms at the transcriptional and post-transcriptional levels, possibly modulated by the hormone environment used during somatic embryogenesis. Nonetheless, the (i) negatively correlated expression of miR160 and *ARF18* in *P. abies* somatic embryogenesis, (ii) the phylogenetic data showing the identity or close relatedness of the miR160 and the target sequences in *Arabidopsis* and conifers, and (iii) the previously gathered degradome data from *P. pinaster* tissues highlighting miR160-*PpARF18* as a high confidence interaction, strongly support the functional interaction during conifer embryo development.

AUXIN RESPONSE FACTOR proteins are key to the transcriptional response to auxin and in *Arabidopsis* they are encoded by a large gene family that acts either as activators or repressors of auxin response genes (Remington et al., 2004; Okushima et al., 2005; Liu et al., 2015). Their biological functions are complex to dissect because different gene family members often show overlapping expression patterns converging on the regulation of specific biological processes (Rademacher et al., 2011). The involvement of different ARFs in several developmental processes controlled by auxins, namely zygotic and somatic embryogenesis regulation is evident in *Arabidopsis* (Rademacher et al., 2011; Wójcikowska and Gaj, 2017). However, their roles in developmental processes in gymnosperms have been less explored. In *Arabidopsis*, at least 14 *ARF* genes are transcribed during SE, including *ARF10*, *ARF16*, and *ARF17* (Wójcikowska and Gaj, 2017) which were described as significantly upregulated during SE induction. In the analyzed *P. abies* SE cultures, the closest stage to SE induction corresponds to proembryogenic masses (PEMI). In this stage, the miR160 target *ARF18* is also highly expressed compared to the subsequent developmental stages. Conifer *ARF18* seems to be actively involved in the early stages of SE, being repressed as the embryo develops to reach maturation. As conifer *ARF18* is closely related to *Arabidopsis ARF16*, as suggested by our phylogenetic analysis (Figure 1D), it likely exhibits auxin response repressor activity which may be important to regulate genes required for active cell proliferation during the induction of SE and early embryo development. A significant accumulation of miR160 in *Arabidopsis* has been reported in somatic embryos (Wójcikowska et al., 2018), but its role has been studied mostly in the induction of SE, where miR160 interaction with the LEC2-mediated pathway was investigated. The authors proposed a model in which the targets of miR160 (*ARF10*/*ARF16*) and of miR165/166 (*PHB*/*PHV*) control SE induction by positively regulating LEC2. This results in the upregulation of *YUC* genes and consequent activation of auxin biosynthesis and accumulation, thus triggering auxin-response genes involved in SE induction (Wójcikowska et al., 2018). LEC2 is also considered a major regulator of seed maturation by controlling the synthesis and the accumulation of protein and lipid reserves (Stone et al., 2008; Wójcik et al., 2017). However, surprisingly, it has been recently reported that *LEC2* is absent from the genomes of *P. abies* and *Pinus taeda* and it is possibly lacking in conifers in general (Ranade and Egertsdotter, 2021), but the availability of additional genomic data from gymnosperm species in the near future, including the genome sequences of *P. pinaster* and other gymnosperms, will help to further clarify this issue. Although the miRNA-mRNA regulatory nodes are thought to have undergone parallel evolution in different plant groups (Cui et al., 2017), the angiosperms and gymnosperm groups diverged approximately 300 million years ago (Magallón et al., 2013). Therefore, alternative or specific regulatory networks may be active where miR160 functions might be involved.

Further molecular studies are also needed to uncover crosstalk of conifer miR160-*ARF18* with other hormone pathways. Indeed, as suggested by Wójcikowska et al. (2018), *ARF10*/*ARF16* may impact the signaling pathways of ABA and/or cytokinins, thereby contributing to SE induction. Dai et al. (2021) also described that the transcript levels of auxin and brassinosteroid signaling-related genes are possibly modulated by miR160-*ARF10*/16/*17* during hypocotyl elongation in *Arabidopsis*.

As far as we know, our study reports the first *in vivo* validation of a gymnosperm miRNA with its predicted target, and the use of this approach as a first step in the functional characterization of miRNAs will be of great utility for future studies of miRNA functions in conifer embryogenesis.

## Data Availability Statement

The original contributions presented in the study are included in the article/Supplementary Material, further inquiries can be directed to the corresponding author.

## Author Contributions

AA, AC, and CM designed the research. AA prepared the constructs, performed the protoplast transfection experiments, and all the expression analyses. PP and BC performed the *in silico* analyses. SL and AM participated in the protoplasts isolation and transfection experiments and RT-PCR analysis, respectively. EB-G, AC, and SC provided guidance during the protoplast transfection experiments and interpretation of results. AA and CM wrote the manuscript. All authors contributed to the article and approved the submitted version.

## Funding

This research was supported by Fundação para a Ciência e Tecnologia, I.P. (FCT), through PhD grants SFRH/BD/128827/2017 to AA, SFRH/BD/143771/2019 to BC, and PD/BD/114359/2016 to SL, CEEC/IND/00175/2017 contract to AM and R&D Unit grants to “GREEN-IT – Bioresources for Sustainability” (UIDB/04551/2020 and UIDP/04551/2020), BioISI (UIDB/04046/2020 and UIDP/04046/2020), and CFE – Center for Functional Ecology – Science for People and the Planet (UIDB/04004/2020). The People Programme (Marie Curie Actions) of the EU Seventh Framework Programme (FP7/2007-2013) is acknowledged for REA grant agreement n° PIEF-GA-2013-627761.

## Conflict of Interest

The authors declare that the research was conducted in the absence of any commercial or financial relationships that could be construed as a potential conflict of interest.

## Publisher’s Note

All claims expressed in this article are solely those of the authors and do not necessarily represent those of their affiliated organizations, or those of the publisher, the editors and the reviewers. Any product that may be evaluated in this article, or claim that may be made by its manufacturer, is not guaranteed or endorsed by the publisher.

## Footnotes

^1^ https://www.mirbase.org

^2^ https://www.ncbi.nlm.nih.gov/refseq/

^3^ Biomers.net

^4^ Congenie.org

^5^ https://primer3.ut.ee
