# Supplementary figures and images for: miR160 Interacts in vivo With Pinus pinaster AUXIN RESPONSE FACTOR 18 Target Site and Negatively Regulates Its Expression During Conifer Somatic Embryo Development
Source: Front Plant Sci. 2022 Mar 15;13:857611. doi: 10.3389/fpls.2022.857611 (PMC8965291; doi:10.3389/fpls.2022.857611)

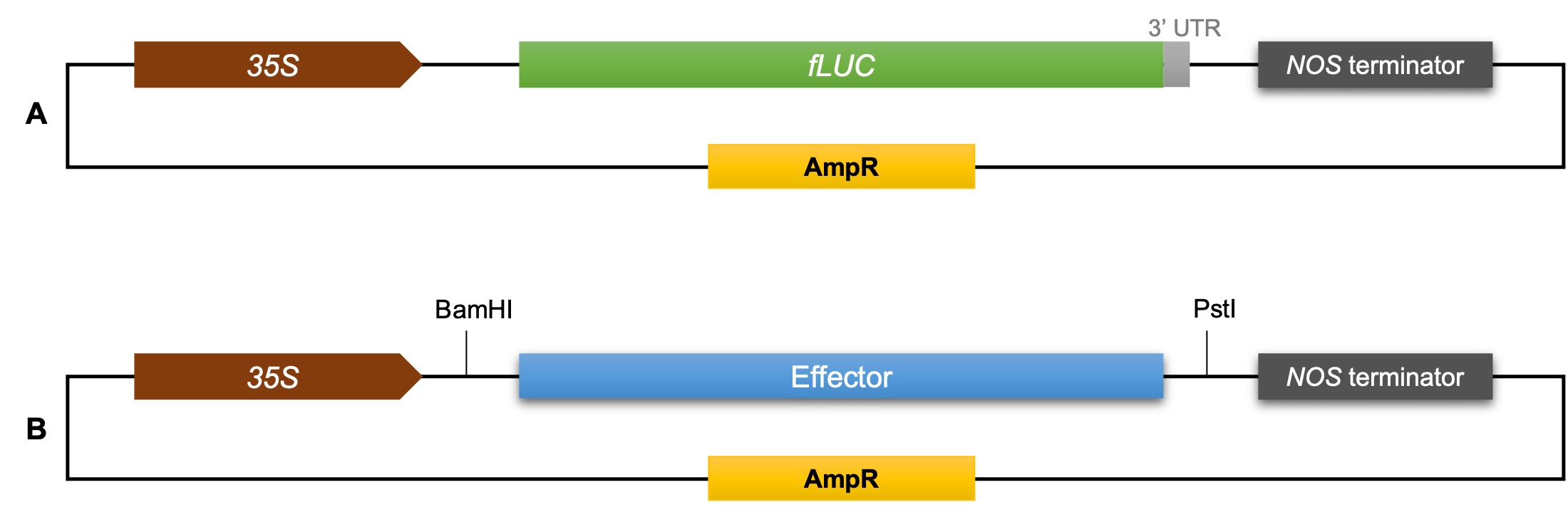

Supplement: Supplementary file 1 [file Image_1.JPEG]
