# Supplementary material for: miR160 Interacts in vivo With Pinus pinaster AUXIN RESPONSE FACTOR 18 Target Site and Negatively Regulates Its Expression During Conifer Somatic Embryo Development
Source: Front Plant Sci. 2022 Mar 15;13:857611. doi: 10.3389/fpls.2022.857611 (PMC8965291; doi:10.3389/fpls.2022.857611)
Supplement: Supplementary file 2 [file Table_1.DOCX]

Supplementary Material

# Supplementary Figures and Tables

## Supplementary Figures

**Supplementary Figure 1.** Schematic pHBT95 vector maps showing the reporter (A) and effector (B) constructs used and the restriction sites. In both cases, the reporter Firefly Luciferase (*fLUC*) and the Effector region are under the control of the *35S* promoter and the *NOS* terminator.

## Supplementary Tables

| **Primer** | **Sequence** |
| --- | --- |
| primiR160_BamHI_F | TTTGGATCCGTTGGTAATGACTTTGTGAG |
| primiR160_PstI_R | AGCCTGCAGCATCAAGAAACTAAGAGGG |
| LUC_UTR_ARF18_F | AAGTCCAAATTGTAAAATAGGCATGCAGGGAGCCAGGCAGTAACTGTATTCAGCGAT |
| LUC_UTR_ARF18_R | ATCGCTGAATACAGTTACTGCCTGGCTCCCTGCATGCCTATTTTACAATTTGGACTT |
| LUC_UTR_NC_ARF18_F | AAGTCCAAATTGTAAAATAGGCATGCAGATAGCCAGGCAGTAACTGTATTCAGCGAT |
| LUC_UTR_NC_ARF18_R | ATCGCTGAATACAGTTACTGCCTGGCTCCCTGCATGCCTATTTTACAATTTGGACTT |
| IPS1_MIM160_A | TTCCGAGGGGAACCGAAGCTTGCCTGGCTCTAGACTGTATGCCATTTCTAGAGGGAGATAAACA |
| IPS1_MIM160_B | GTTTATCTCCCTCTAGAAATGGCATACAGTCTAGAGCCAGGCAAGCTTCGGTTCCCCTCGGAA |
| RT_pabARF18_F | TCATGAAGACTGTGAGGAGGC |
| RT_pabARF18_R | GGAACATGAAGCCAACAAGATG |

**Supplementary Table 1.** List of primers.
